# Books and Pamphlets Received

**Published:** 1858-11

**Authors:** 


*Books and Pamphlets Received.*—Several books are on our table waiting for space for review, which we have already acknowledged. We have since received:

*Observations on Malarial Fever.* By Joseph Jones, A. M., M. D., Prof. of Chemistry and Pharmacy in the Medical College of Georgia, Augusta, etc., etc.

*Electrical Anæsthesia:* comprising a brief History of the Discovery, also full Directions for its Application in Surgical and Dental Operations. Dedicated (by permission) to Dr. Frank H. Hamilton, Professor of Surgery in the Buffalo Medical College. By W. G. Oliver.

*West on Diseases of Women.* Part II.; and *Morland on the Urinary Organs.* From Blanchard & Lea.

*Uræmic Convulsions.* By Braun, of Vienna; and *Nature and Art in Disease.* By Sir J. Forbes. From S. S. & W. Wood.

*Transactions of the New Hampshire State Medical Society.*

*Transactions of the Illinois State Medical Society for the Year* 1857.

We have seen favorable notices of *Bennett's Clinical Lectures*, issued by the same house, a work which we have not yet received.

The above works from the distinguished reputation of the authors, demand a careful attention, which we will bestow upon them at the earliest opportunity.

*A New Imponderable Agent.*—We make the following extract from the "Medical and Surgical Reporter," in which the author professes to have discovered a new imponderable agent. This communication is exceedingly curious; but we must certainly wait farther developments for the confirmation of these discoveries. Some years ago, Baron Reichenback published a monograph, entitled "Researches into Odylic Phenomena." He then described a new dynamid, under the name of "Od," which exerted its force in various ways, such, for example, as making a button suspended by a thread, held in the hand of a person, strike the correct hour of the day, independently of the volition of the individual; or determining the direction of the divining rod. Most physiologists, however, explained these phenomena by the theory of the involuntary and unconscious action of the muscles, while the subject was in a state of "expectant attention." The following letter seems to be a continuation of these researches; we present it to our readers as a curious statement, the value of which, however, we are by no means prepared to estimate:

*Researches into Odic Phenomena: Discovery of A New Cosmal Agent.* From Letters of Baron Reichenbach, of Vienna. Extracted and Translated by Dr. G. Bachman.

Baron Reichenbach, of Vienna, recently published the results of his experiments on sensitive persons of marked nervous temperament, who are capable of perceiving the manifestation of an imponderable agent, hitherto unknown, and unappreciable by persons not endowed with a nervous temperament. The Baron gives to this agent the name of *Od.* He refers to a future one of the series of letters in which he publishes his observations, to explain the etymology of the term.

The first peculiar phenomenon, which was perceived by a lady of his acquaintance, and which led him to experiment further, is this: A large mountain-crystal, which had a flat base and pointed apex, was lying horizontally across the corner of a table, projecting at its two ends. The room was completely dark, which darkness was broken by a blue light, emanating from the sharp end, in an undulating, scintillating motion, and a yellow flame issuing, with a similar movement, from the blunt end of the crystal. On subsequent experiments with hundreds of other sensitive persons, as he calls them, he found this remarkable phenomenon confirmed, and also found that these persons, on approaching the crystal with the left hand within three inches, or so many feet, (if the crystal be a large one,) would experience a cool breath issuing from the sharp end, and a lukewarm one from the base end. The former is accompanied by a sensation of agreeable, exhilarating freshness; the latter, by repugnance, and sometimes nausea. The Baron, startled at these singular manifestations, was at a loss to refer them to any known physical and physiological laws. It cannot be heat, for there is no source of heat, and the thermoscope remains unaffected by it. Electricity it is not, for there is no electro-excitor at play; the electroscope remains, also, unaffected by it, and conduction according to electrical laws, is fruitless.

Magnetism it cannot be, for crystals are not magnetical; nor can it be light, as mere light is not attended by sensations of warmth or cold.

The Baron being convinced of having discovered a new dynamid, carried his investigations further, and next tested its effects and presence in the sun. He placed the observer in the shade, holding a glass rod, or even wooden stick in the left hand, ordering the rod or stick to be held in the light of the sun. According to our received notions of conduction and radiation of heat, we should expect to hear the observer say that a sense of heat in his hand was the result of the test, but the contrary takes place. The hand will feel cool, and immediately on retracting the rod into the shade, a sensation of heat will follow.

These are the circumstances which reverse the order of things as perceived until now,—the rays of the sun do produce in certain conditions just what they are expected to obviate, cold—and this cold, the subjects perceiving it will tell you, has an effect analogous to that which that issuing from the pointed crystals manifests. If this phenomenon just described is of the nature of the *od*, it will, like the crystal, emanate light in the darkness. Thus sylogizing, the Baron conducted a copper wire from the light of the sun into a darkened room, in which representatives of the temperament mentioned heretofore were posted. Immediately the persons belonging to that class observed the wire glowing, and a flame of the size of a finger rising from its end. Go a step further, and throw the colors of the rainbow through a glass prism, on the next wall, let the nervous individual catch the reflected colors by means of the glass or wooden rod, on the mere hand; the effect will be as follows: The blue color will create a pleasant, refreshing sensation, more so than from the light of the sun, the yellow will produce the known lukewarmness of the odic tests already mentioned, and the red will increase the latter effects and fatigue the arm. This seems to furnish an index at the same time, that caprices which individuals show in regard to color, may have another cause than mere optical impression. Aside from the odic effects of the colors, the Baron proves the *od* nature of the solar rays. Polarize them in the known manner, that you let them pass through a bundle of a dozen glass discs, on an angle of 35 deg.; then let the sensitive observer throw the rod alternately into the direct and reflected light, and the result will be an alternate sense of cold and lukewarmness, as the rod passes the direct or reflected light.

Further: place a glass of water under the direct or reflected light, or under the blunt end and the sharp end of the crystal, or under the blue color of the artificial iris and the yellow of the same, and that which produces coolness of the reägents cited, will acidulate the water, after being from five to ten minutes under its influence, and that which produces the lukewarmness will cause the water to taste bitter and nauseating to the sensitive, while the former he will drink with pleasure, and think it refreshing.

The experiments in respect to the solar light will be polarily reversed if the moonlight is experimented on.

The Baron promises to show, in his future letters, what the influence of his discovered cosmic dynamid is on the entire universe.

